# Lobomycosis in Amazon Region, Bolivia, 2022

**DOI:** 10.3201/eid3012.241089

**Published:** 2024-12

**Authors:** Maria I. Méndez, Rony Colanzi, Jose A. Suárez, Homero Penagos, Carolina Hernandez, Ruth Garcia-Redondo, Juan D. Ramirez, Alberto Paniz-Mondolfi

**Affiliations:** Centro Nacional de Enfermedades Tropicales, Santa Cruz, Bolivia (M.I. Méndez); Laboratorio Catedral, Santa Cruz (R. Colanzi); Universidad Internacional Sek Quito, Quito, Ecuador (J.A. Suárez); Universidad Autónoma de Chiriquí, Chiriquí, Panama (H. Penagos); Icahn School of Medicine at Mount Sinai, New York, New York, USA (C. Hernandez, R. Garcia-Redondo, J.D. Ramirez, A. Paniz-Mondolfi)

**Keywords:** lobomycosis, *Paraccocidiodes loboi*, *Lacazia loboi*, fungi, Andes, Amazon, Bolivia

## Abstract

We report a patient with lobomycosis caused by *Paracoccidioides loboi* fungi in the Andes-Amazon region of Bolivia. We examined clinical, epidemiologic, and phylogenetic data and describe potential transmission/environmental aspects of infection. Continued surveillance and identification of lobomycosis cases in South America are crucial to prevent the spread of this disease.

Lobomycosis, also known as lacaziosis, is a rare chronic fungal infection primarily involving the skin and subcutaneous tissues caused by the dimorphic Onygenales fungus *Paracoccidioides loboi* (also known as *Lacazia loboi*). It is endemic to Central and South America, particularly within the Amazon rainforest, although its precise geographic boundaries remain poorly delineated and appear to be expanding (*1*). The first documented case of lobomycosis in Bolivia was reported in 1982 (*2*). Since then, 2 additional cases have been reported, distributed across the department of Pando in Bolivia in close proximity to Brazil (*3*).

Despite an increasing number of case reports, gaps persist in understanding the eco-epidemiologic and biogeographic aspects of lobomycosis, especially across the vast regions of the Andes. We report a patient from Cobija, located in the Pando Department of Bolivia, who had lobomycosis caused by *P. loboi*. We also describe the epidemiology and phylogeography of lobomycosis and the need for continued research to determine the geographic reach of this fungal infection across regions of the Andes in South America.

## The Study

A 71-year-old man, originally from Cobija in northern Bolivia, sought care at Centro Nacional de Enfermedades Tropicales (CENETROP) in Santa Cruz, Bolivia, in December 2022. He reported longstanding cutaneous lesions on his left ear, associated with pruritus and a chronic, insidious headache. He recalled that the lesions began when he was 11 years of age after he was bitten by a mosquito while playing in the Arroyo Virtudes river in his hometown in Pando Department, Bolivia. The lesions slowly progressed from single nodules to a confluent, enlarged nodular plaque. Despite multiple consultations with local shamans and healthcare providers, including 2 instances of surgical removal by otolaryngologists for presumed pinna perichondritis, a precise etiologic diagnosis remained elusive, and the patient remained untreated for 60 years with invariable recurrence of lesions.

**Figure 1:**
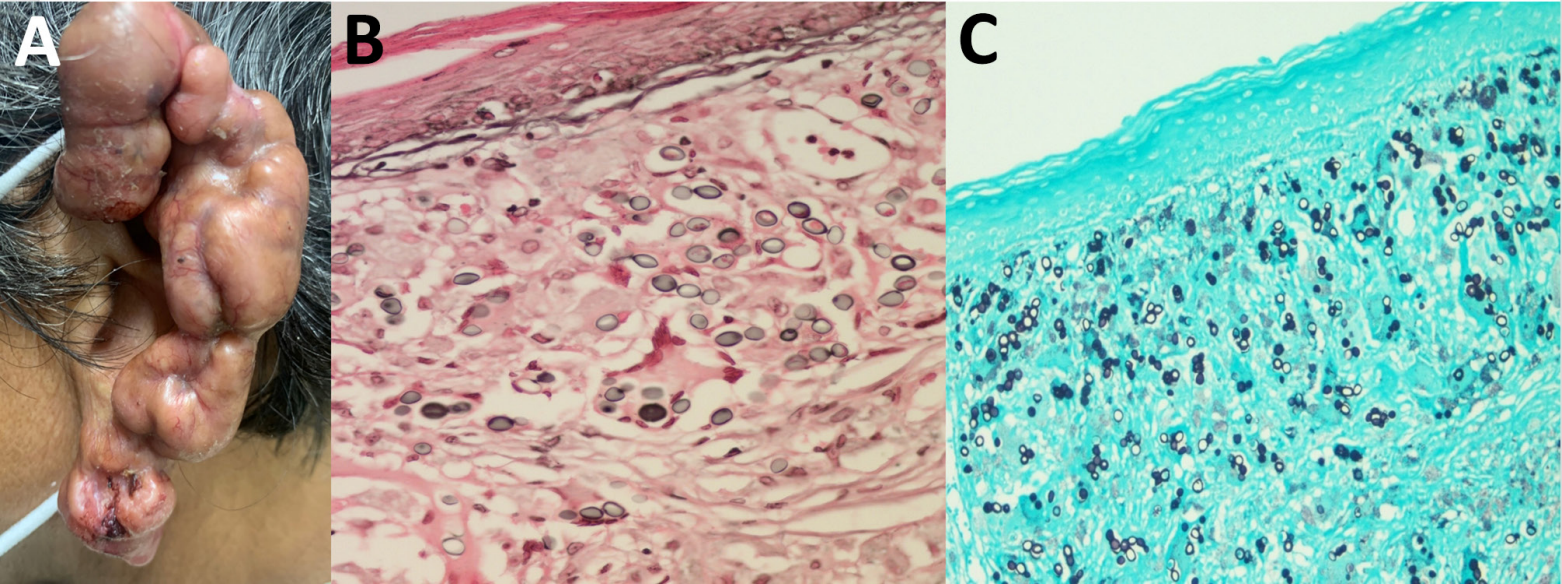
Clinical manifestations of lesions in patient with lobomycosis in Amazon Region, Bolivia, 2022. A) Multiple hyperpigmented, confluent keloid-like nodular lesions with overriding telangiectasias and patchy crusted and eroded squamous areas were noted on the helix and lobule of the left ear. B, C) Histologic examination of lesion specimens showed oval-to-round shaped cells that had connecting tubular projections organized in a string-of-pearls pattern, in pairs and individually. B) Fontana-Masson stain; original magnification ×1,000. C) Gömöri methenamine silver stain; original magnification ×400.

Physical examination revealed that the entire left ear was replaced by keloid-like confluent nodular lesions, which appeared infiltrated and hyperpigmented with overriding telangiectasias (Figure 1, panel A). Patchy squamous crusted and eroded areas were noted on the helix and lobule. No noticeable lymphadenopathy existed, and the rest of the physical examination was unremarkable. Differential diagnoses included diffuse cutaneous leishmaniasis, leprosy, sporotrichosis, chromoblastomycosis, dermatofibrosarcoma protuberans, dermatofibroma, sarcomas, and cutaneous tuberculosis. We performed a biopsy, and histopathologic examination revealed abundant lemon-shaped, refractile, yeast-like cells that were single and in chains within a background of granulomatous inflammation, consistent with *P. loboi* infection (Figure 1, panels B, C).

**Figure 2:**
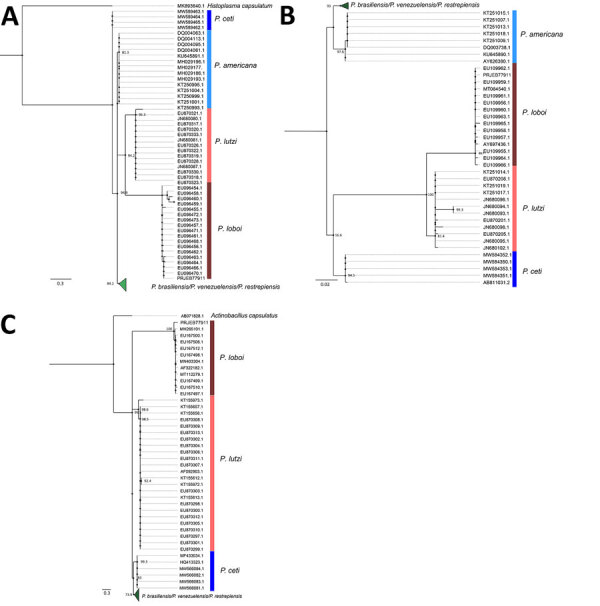
Phylogenetic analysis of genes from different *Paracoccidioides* spp. in study of lobomycosis in Amazon Region, Bolivia, 2022. Phylogenetic trees were inferred by using the maximum-likelihood method for *ADP-rf* (A), *GP43* (B), and *ITS1*/*ITS2* (C) genes. Sequences obtained from GenBank were compared with sequences from the patient sample (sequences deposited in European Nucleotide Archive [https://www.ebi.ac.uk/ena] under Bioproject no. PRJEB77911). Scale bars indicate nucleotide substitutions per site.

We extracted DNA from the formalin-fixed paraffin-embedded biopsy tissue sample. We used PCR and Oxford nanopore sequencing (Oxford Nanopore Technologies, https://nanoporetech.com) to detect and identify *ITS1*, *ITS2*, *ADP-rf*, and *GP43* genes (*4*). We compared the sequences to a reference sequence dataset of various *Paracoccidioides* spp. from GenBank. We reconstructed a phylogenetic tree by using the maximum-likelihood method and IQ-TREE 2 (http://www.iqtree.org) and assessed node support by using bootstrap (1,000 replicates), abayes, and SH-aLRT methods in IQ-TREE (Figure 2). We selected substitution models according to the Bayesian information criterion. We deposited DNA sequences in the European Nucleotide Archive (https://www.ebi.ac.uk/ena; project no. PRJEB77911; submission no. ERA30711349).

The patient underwent treatment with itraconazole (200 mg/d for 3 months). After the first month of treatment, he experienced symptomatic improvement of the headache and resolution of ear pruritus, although the ear lobe deformation persisted. After 3 months of treatment, the patient chose to stop the medication because of intolerable gastric side effects and opted for surgical resection.

Lobomycosis is endemic in many Latin America countries; most cases have been reported from Brazil (*5*). However, the disease has also been reported across Andes regions in Venezuela, Colombia, Ecuador, Peru, and Bolivia (*3*). Limited knowledge exists about endemicity and ecologic factors driving disease occurrence in the Andes regions. Lobomycosis was first reported in Bolivia in 1982 in a 58-year-old farmer who had skin nodules on his earlobe, face, and extremities, and a diagnosis of lobomycosis was confirmed by direct smear and histological examination of lesions. That patient was treated with a combination of pharmacological agents, including amphotericin, flucytosine, and ketoconazole, along with surgical treatment, and invariable recurrence was observed (*2*). Since 1982, only 2 more cases of lobomycosis have been reported from Bolivia in the literature (*3*); those cases occurred in the department of Pando near the Amazon Region of Brazil.

*Paracoccidioides* spp., similar to many dimorphic fungi, usually exhibit geographic restrictions linked to specific environmental conditions (*6*). Although soil and vegetation are believed to be the primary habitats of *P. loboi*, human infections have also been associated with proximity to water, suggesting that *P. loboi* might be a hydrophilic microorganism (*1*). *P. ceti* is known to infect dolphins (*7*), *P. braziliensis* infections have been reported in fish (*8*), and the recently described *Emydomyces testavorans* has been reported to cause shell lesions in fresh water chelonians (*9*). *P. loboi* and other fungi in the order Onygenales exhibit ecologic traits that have shaped their evolutionary, ecologic, and pathogenic characteristics, enabling them to thrive in complex habitats (*10*). Thus, conditions such as wet soil and high acidity, which are commonly found in the tropical Andes regions bordering the Amazon rainforest, appear to be suitable niches for *P. loboi*.

Because of the high biodiversity of the Andes and the association of *P. loboi* fungus with aquatic and sylvatic environments (*1*), cases of lobomycosis might be substantially underreported in countries with Andes mountain regions, particularly those sharing territories overlapping the Amazon and regions of the broader Andes-Amazon basin. By 2022, a total of 907 cases of lobomycosis had been reported worldwide (*5*). In the Andes regions, the total number of lobomycosis cases was 94, and a clear geographic trend toward occurrence eastward at the Andes-Amazon piedmont was observed for 84 of those cases. In contrast, the western regions accounted for only ≈10 cases (Appendix Table 1). Of the total number of cases reported worldwide, 496 (55%) cases have been reported from Acre State in Brazil (Appendix Table 2) (*5*). Together with the departments of Madre de Dios (Peru) (*11*) and Pando (Bolivia) (*2*), where this case occurred, those 3 regions form a tri-border area harboring most lobomycosis cases in each country, making it a disease-endemic hotspot. Moreover, the stream where the patient recalled being exposed to the infection as a child is a tributary of the Acre River, the main waterway within this tri-border area. The patient recalled a mosquito bite during his childhood, which raises the longstanding question about the potential transmission of this fungus through aquatic fauna, such as copepods, snails, or other aquatic insects (*12*). This topic warrants further research to decipher potentially unknown transmission mechanisms.

## Conclusions

Lobomycosis cases have been reported in countries such as Colombia that are northwest of the Andes Mountain range (*13*), suggesting that geographic ranges of endemicity in the tropical Andes and Amazon might be expanding. Expansion might be occurring in other biogeographic regions northward into Central America through the moist tropical and deciduous forests of the Chocó-Darien region into Panama, where 1 case was reported in 2022 (*14*). Understanding ecologic niches and environmental variability is crucial for preventing transmission of infections caused by *P. loboi*. Ongoing climatic changes might also exert environmental pressures that lead to *P. loboi* emergence or spillover beyond known areas of disease endemicity. Continued surveillance and correct identification of lobomycosis cases in the Andes region and other parts of South and Central America are crucial to prevent the spread of this disease.

Appendix Additional information for lobomycosis in Amazon Region, Bolivia, 2022.
