# Supplementary material for: Lobomycosis in Amazon Region, Bolivia, 2022
Source: Emerg Infect Dis. 2024 Dec;30(12):2660–3. doi: 10.3201/eid3012.241089 (PMC11616652; doi:10.3201/eid3012.241089)
Supplement: Appendix — Additional information for lobomycosis in Amazon Region, Bolivia, 2022. [file 24-1089-Techapp-s1.pdf]

Article DOI: https://doi.org/10.3201/eid3012.241089
EID cannot ensure accessibility for supplementary materials supplied by authors. Readers who have difficulty accessing supplementary content should contact the authors for assistance.

Lobomycosis in Amazon Region, Bolivia, 2022

Appendix

Appendix Table 1. Cases reported in the Andes region*

Country	No. cases	References
Venezuela	27	(*1–4*)
Colombia	59	(*4–8*)
Ecuador	2	(*4*)
Peru	3	(*9*)
Bolivia	3	(*4*)
Total no. cases	94	NA

*NA, not applicable.

Appendix Table 2. Worldwide cases of lobomycosis*

Country	No. cases	Potential exposure/contributing factors	References
Venezuela	27	NA	(*1–4*)
Colombia	59	NA	(*4–8*)
Ecuador	2	NA	(*4*)
Peru	3	NA	(*9*)
Bolivia	3	NA	(*4*)
India	1	No travel history; past medical history of COVID-19	(*10*)
Greece†	1	Agriculture worker with no travel history; past medical history of common variable immunodeficiency	(*11*)
South Africa†	2	Case 1 travel history to Mexico; case 2 was swimmer/scuba diver with travel history to Palestine and United Kingdom	(*12*)
Panama	14	NA	(*4,13*)
Mexico	2	NA	(*4,14*)
Canada†	1	Travel history to Guyana and Venezuela	(*15*)
United States†	1	Travel history to Venezuela	(*16*)
Brazil	496	NA	(*17*)
Costa Rica	21	NA	(*4*)
Suriname	34	NA	(*4*)
French Guiana	31	NA	(*18*)
Guyana	4	NA	(*4*)
Spain†	1	NA	(*17*)
France†	1	NA	(*17*)

*NA, not applicable.
†Lobomycosis nonendemic countries.

References

1. Beltrame A, Danesi P, Farina C, Orza P, Perandin F, Zanardello C, et al. Case report: molecular confirmation of lobomycosis in an Italian traveler acquired in the Amazon Region of Venezuela. Am J Trop Med Hyg. 2017;97:1757–60. PubMed https://doi.org/10.4269/ajtmh.17-0446

2. Bermudez L, Van Bressem MF, Reyes-Jaimes O, Sayegh AJ, Paniz-Mondolfi AE. Lobomycosis in man and lobomycosis-like disease in bottlenose dolphin, Venezuela. Emerg Infect Dis. 2009;15:1301–3. PubMed https://doi.org/10.3201/eid1508.090347

3. Paniz-Mondolfi AE, Reyes Jaimes O, Dávila Jones L. Lobomycosis in Venezuela. Int J Dermatol. 2007;46:180–5. PubMed https://doi.org/10.1111/j.1365-4632.2007.02937.x

4. Brito AC, Quaresma JAS. Lacaziose (doença de Jorge Lobo): revisão e atualização. An Bras Dermatol. 2007;82:461–74. https://doi.org/10.1590/S0365-05962007000500010

5. Arenas CM, Rodriguez-Toro G, Ortiz-Florez A, Serrato I. Lobomycosis in soldiers, Colombia. Emerg Infect Dis. 2019;25:654–60. PubMed https://doi.org/10.3201/eid2504.181403

6. Cabrera-Salom C, González LF, Rolón M, Sánchez BF. Keloids on the ears. Int J Dermatol. 2017;56:819–21. PubMed https://doi.org/10.1111/ijd.13556

7. Cataño JC, Londoño N. A keloid-like tumour. Eur J Intern Med. 2024;120:116–7. PubMed https://doi.org/10.1016/j.ejim.2023.12.004

8. Ihara GM, Massone C, Schettini AP, Maroja MF. Leprosy and lobomycosis: first report from the Amazon Region. Lepr Rev. 2015;86:195–201. PubMed https://doi.org/10.47276/lr.86.2.195

9. Ramírez Soto MC, Malaga G. Subcutaneous mycoses in Peru: a systematic review and meta-analysis for the burden of disease. Int J Dermatol. 2017;56:1037–45. PubMed https://doi.org/10.1111/ijd.13665

10. Chavan SS, Chandrashekhar TN. Lobomycosis in a post-Covid 19 patient: a case report and review of literature. Turk Patoloji Derg. 2023;39:206–11. PubMed https://doi.org/10.5146/tjpath.2023.01604

11. Papadavid E, Dalamaga M, Kapniari I, Pantelidaki E, Papageorgiou S, Pappa V, et al. Lobomycosis: a case from southeastern Europe and review of the literature. J Dermatol Case Rep. 2012;6:65–9. PubMed https://doi.org/10.3315/jdcr.2012.1104

12. Al-Daraji WI, Husain E, Robson A. Lobomycosis in African patients. Br J Dermatol. 2008;159:234–6. PubMed https://doi.org/10.1111/j.1365-2133.2008.08586.x

13. Suárez JA, Cerrud B, Pachar M, Patiño LH, Reidy J, Chace A, et al. Human lobomycosis caused by *Paracoccidioides* (*Lacazia*) *loboi*, Panama, 2022. Emerg Infect Dis. 2023;29:2513–7. PubMed https://doi.org/10.3201/eid2912.231092

14. Pech-Ortiz L, Maya-Aranda S, Hernández-Castro R, Xicohtencatl-Cortes J, Tirado-Sánchez A, Bonifaz A. Lacaziosis (lobomycosis) from southern Mexico: a case confirmed by molecular biology. Mycopathologia. 2020;185:737–9. PubMed https://doi.org/10.1007/s11046-020-00464-7

15. Elsayed S, Kuhn SM, Barber D, Church DL, Adams S, Kasper R. Human case of lobomycosis. Emerg Infect Dis. 2004;10:715–8. PubMed https://doi.org/10.3201/eid1004.030416

16. Burns RA, Roy JS, Woods C, Padhye AA, Warnock DW. Report of the first human case of lobomycosis in the United States. J Clin Microbiol. 2000;38:1283–5. PubMed https://doi.org/10.1128/JCM.38.3.1283-1285.2000

17. Gonçalves FG, Rosa PS, Belone AFF, Carneiro LB, de Barros VLQ, Bispo RF, et al. Lobomycosis epidemiology and management: the quest for a cure for the most neglected of neglected tropical diseases. J Fungi (Basel). 2022;8:494. PubMed https://doi.org/10.3390/jof8050494

18. Grotta G, Couppie P, Demar M, Drak Alsibai K, Blaizot R. Fungal density in lobomycosis in French Guiana: a proposal for a new clinico-histological and therapeutic classification. J Fungi (Basel). 2023;9:1005. PubMed https://doi.org/10.3390/jof9101005
